# Research roadblocks: a cross-sectional study on barriers and perceptions among medical students in Sudan

**DOI:** 10.1186/s12909-025-07632-7

**Published:** 2025-07-11

**Authors:** Elnazeir Mohamed Ibrahim Mohamedzain, Muhannad Bushra Masaad Ahmed, Mohamed Salah Abdulrazeg Abdulrahman, Bakhiet Ahmed Abaker Suliman, Mohammed Elturabi Ajib Alabas, Alam Eldeen Hamid Omer Osman, Yousif Abdelrahman Yousif Fadlelmoula, Azmy Albadry Abdeen Babiker, Ahmed Balla M. Ahmed, Mohanad Altayeb Mokhtar Gobbara, Sohaib Mohammed Mokhtar Ahmed

**Affiliations:** 1 Faculty of Medicine and Health Sciences, University of Gadarif, Gadarif, Sudan; 2 Faculty of Medicine, Alzaiem Alazhari University, Khartoum, Sudan; 3 Research Office, Flame of Hope Initiative, Gadarif, Sudan; 4 Faculty of Medicine, University of Khartoum, Al-Qasr Street, PO Box: 102, Khartoum, 11111 Sudan

**Keywords:** Research barriers, Knowledge, Attitudes, Practices, Healthcare research, Medical students, Sudan

## Abstract

**Background:**

Medical research is essential for improving healthcare, yet medical students in Sudan face significant barriers to conducting research. These obstacles, compounded by Sudan’s political unrest and limited resources, hinder students’ engagement and contributions to evidence-based practice. This study aimed to evaluate the knowledge, attitudes, practices, and perceived barriers toward research among undergraduate medical students in Sudan.

**Methods:**

A cross-sectional study was conducted across public and private medical colleges in Sudan. Data were collected from 1123 students using an online questionnaire from November to December 2023. The standardized survey measured demographics, knowledge, attitudes, research practices, and perceived barriers. Descriptive statistics were analyzed using SPSS version 27. Chi-square test, Mann-Whitney U test, Kruskal-Wallis test, and Spearman’s rank correlation were used for group comparisons and assessing associations. Significance was determined at p-value < 0.05.

**Results:**

The majority of students (92.0%) exhibited poor knowledge of research, with a mean score of 1.2/8. However, students demonstrated positive attitudes, with a mean score of 69.2/93. 50% of students had no prior research experience, and 55.2% had not attended research methodology workshops. Key barriers included lack of funding (67.6%), insufficient access to databases (58.1%), and inadequate laboratory equipment (64.8%).

**Conclusion:**

While medical students in Sudan hold positive attitudes toward research, their limited knowledge and practical engagement highlight the need for improved research education. Addressing barriers such as financial constraints and inadequate resources is essential for fostering a research-oriented culture and enhancing students’ contributions to healthcare research in Sudan.

## Background

Advancement in healthcare relies heavily on continuous medical research, which drives innovation in disease prevention, diagnosis, and treatment. The foundation of effective clinical practice is built upon robust scientific evidence, making research a vital component of high-quality medical care. For physicians, staying up-to-date with emerging evidence and actively engaging in research is essential to ensure informed decision-making and improved patient outcomes [1].

In addition to its clinical importance, research fosters critical thinking, analytical skills, and clinical reasoning among healthcare professionals, especially during their formative training years [2]. Despite its significance, many medical students encounter substantial barriers that hinder their involvement in research. These include insufficient mentorship, competing academic and extracurricular demands, and limited training in research methodology [3–5]. Such challenges are even more pronounced in low-income countries, where access to research infrastructure, funding, and training opportunities remains limited [5, 6]. The global disparity in research capacity between high- and low-income nations is evident, with many resource-limited settings experiencing a decline in active researchers. This is largely due to systemic constraints that undermine local research productivity and impede the development of a sustainable research culture [7].

In Sudan, medical research faces additional challenges including political instability, brain drain, and the ongoing impact of war [8]. Undergraduate students often graduate without essential research skills due to heavy curricular demands, limited methodological exposure, and insufficient time. While some reforms have aimed to incorporate research into medical education, progress is hampered by inadequate infrastructure, a shortage of trained faculty, and limited funding [9, 10]. A recent study at a Sudanese university found that 91.3% of medical students acknowledged the importance of research, and 76% supported its inclusion in the curriculum. However, only 59.6% believed it should be a requirement for the MBBS degree, and just 63.5% considered it essential for residency training. The study emphasized the need for curriculum reforms and recommended small group learning models to improve supervision and address barriers such as time constraints and funding shortages [5].

Although previous studies in Sudan, including the one cited, have explored medical students’ perceptions of research, they were generally limited to single institutions or specific regions, restricting the generalizability of their conclusions. Moreover, these studies primarily focused on either perceptions or barriers, often overlooking students’ actual engagement in research activities. This study addresses these limitations by employing a nationally representative sample from universities across Sudan and by assessing both research knowledge and practical involvement—dimensions that have rarely been examined together in this context. The findings offer a more comprehensive basis for developing targeted curriculum reforms that reflect the realities of Sudan’s resource-limited academic environment.

This study aimed to assess the knowledge, attitudes, practices, and perceived barriers to research among undergraduate medical students across Sudanese universities, providing insights into the current state of research education and proposing recommendations to foster a more research-oriented academic culture.

## Methods

### Study design and setting

This cross-sectional study was conducted in public and private medical colleges throughout Sudan to identify and analyze the barriers faced by medical students in conducting research. The data were collected from November 1 to December 31, 2023.

### Study population

Participants were undergraduate medical students who were 18 years or older, enrolled in Sudanese public or private medical schools. The sample included students from all medical schools across Sudan, ensuring national representation. Eligible participants had access to a smartphone or laptop with internet connectivity and provided voluntary electronic informed consent at the beginning of the questionnaire. Those who did not provide consent were excluded from participation.

### Sampling

We determined the sample size using Cochrane’s formula, given the lack of official data on the number of medical students in Sudan. With an assumed population proportion of 50%, a 5% margin of error, and a 95% confidence level, the minimum required sample size was calculated at 385 participants. To enhance the study’s validity and generalizability, it was intended to approach as many students as possible to gather maximum data during the data collection period. The final sample size reached was 1123 medical students. Convenience sampling was employed in this study due to the challenges posed by the ongoing conflict in Sudan, which made accessing official student records impossible. The war has disrupted institutional operations, including data management systems, resulting in the unavailability of updated, centralized records of medical students.

### Data collection instrument

A Google Forms-based online survey was distributed via widely used social media platforms, including WhatsApp, Telegram, and Facebook. These platforms were especially relevant during the ongoing conflict in Sudan, which has driven much of the education online. The survey, which took approximately eight minutes to complete, was anonymous, and no incentives were offered. The questionnaire was prepared in English, which poses no challenges, as English is the primary language of instruction in Sudanese medical colleges [11]. The study employed a previously validated questionnaire adapted from research conducted across six Arab countries, including Sudan [12]. Permission to use and adapt the questionnaire was obtained from the original authors. A separate pre-study was not conducted, as the tool had already been validated in comparable populations, and its content, length, and format were considered appropriate for our context. The survey was divided into five sections to measure the following variables:

#### Demographics

This section collected information on participants’ sex, age, type of university (public or private), academic year, and whether they had studied research methodology at their institution.

#### Knowledge assessment

Participants answered eight multiple-choice questions designed to test their knowledge of basic research concepts, with an option for “I don’t know”. The questions were adapted from Memarpour et al. [13], with the original scale demonstrating a Cronbach’s alpha of 0.71. Responses were scored, with correct answers receiving one point and incorrect or “I don’t know” responses receiving zero. Scores ranged from 0 to 8, with knowledge levels categorized as poor (below 50%), fair (50–75%), or satisfactory (above 75%) [12].

#### Attitude assessment

This section consisted of 31 items measured on a three-point Likert scale (Agree, Uncertain, Disagree), focusing on five dimensions: the perceived usefulness of research (nine questions), research-related anxiety (eight questions), positive attitudes toward research (seven questions), relevance of research to life (four questions), and perceived research difficulty (three questions). The scale used was adapted from the “attitudes toward research scale” [14], with the original study reporting Cronbach’s alpha values for its subscales ranging from 0.71 to 0.92. The overall scores varied across different factors: 3–27 for research usefulness, 3–24 for research anxiety, 3–21 for positive attitudes, 3–12 for relevance, and 3–9 for research difficulty. Higher scores indicated more positive attitudes in each category.

#### Research practices

Participants were asked eight questions reproduced from Alghamdi et al. [4] about their involvement in research. These included yes/no questions about attending research workshops and participating in research projects, followed by questions about the number of projects, publications, oral presentations, and posters. Two additional questions addressed the type of research and the roles students took on during the research process.

#### Barriers to research

This section assessed 32 potential barriers to research participation reproduced from Memarpour et al. [13], with responses measured on a three-point Likert scale (Agree, Uncertain, and Disagree). The original scale reported a Cronbach’s alpha of 0.88.

### Data management

The data were analyzed using SPSS software, version 27. Descriptive statistics, including means, standard deviations, frequencies, and percentages, were employed. These statistics were used to summarize the demographic characteristics, as well as the participants’ knowledge, attitudes, practices, and perceived barriers. The Mann-Whitney U test was employed to compare knowledge, attitudes, and perceived barriers across different groups, such as gender, university type, and research methodology course attendance. The Chi-square test was used to assess associations between categorical variables, while the Kruskal-Wallis test compared non-normality distributed continuous variables across multiple groups. Spearman’s rank correlation was used to assess the relationship between age, knowledge, and barriers. Significance was determined at p-value < 0.05.

## Results

### Sample description

Out of the 1123 students surveyed, 70.3% were female, with a mean age of 22.6 years. Regarding university type, 42.7% were enrolled in private universities, while 57.3% attended public institutions. In terms of academic level, 9.1% were first-year students, 16.6% were in their fifth year, and 17.3% were in their sixth year. Additionally, 56.5% reported having received instruction in research methodology during their university education (Table 1).

**Table 1:** Socio-demographic characteristics of the participants

Characteristic	*N* (%)
**Sex**	
Female	789 (70.3%)
Male	334 (29.7%)
**Age**	
Mean (SD)	22.6 (2.7)
**Type of university**	
Private	480 (42.7%)
Public	643 (57.3%)
**level of study**	
First year	102 (9.1%)
Second year	234 (20.8%)
Third year	233 (20.7%)
Fourth year	174 (15.5%)
Fifth year	186 (16.6%)
Sixth year	194 (17.3%)
**Did you study research methodology in your university?**	
No	488 (43.5%)
Yes	635 (56.5%)

### Students’ knowledge about research

The students had a mean knowledge score of 1.2 out of 8 (SD = 1.4), reflecting a generally poor level of research knowledge. When categorized, the majority of students—1033 (92.0%)—demonstrated poor knowledge, followed by 89 (7.9%) who exhibited a fair level, while only 1 (0.1%) student achieved a satisfactory level.

### Students’ attitudes toward research

The mean total score on the attitude scale was 69.2 out of 93, reflecting a moderately positive overall attitude toward research. Students perceived research as moderately challenging, with a mean difficulty score of 5.1 out of 9. Despite this, they recognized its relevance to daily life, as shown by a high mean score of 8.5 out of 12. Levels of research-related anxiety varied, with an average score of 14.0 out of 24. Notably, students’ positive attitude toward research is evident, with a mean score of 17.6 out of 21. Furthermore, they expressed a strong belief in the usefulness of research for their future careers, evidenced by a mean score of 23.9 out of 27.

### Students’ practices of research

**Fig. 1:**
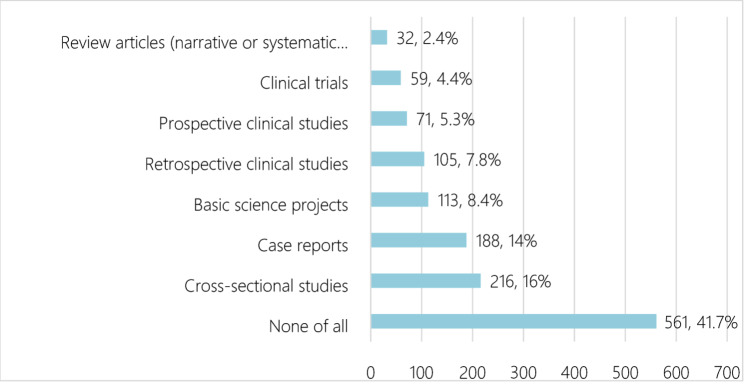
The most common type of studies that the students participated in

**Fig. 2:**
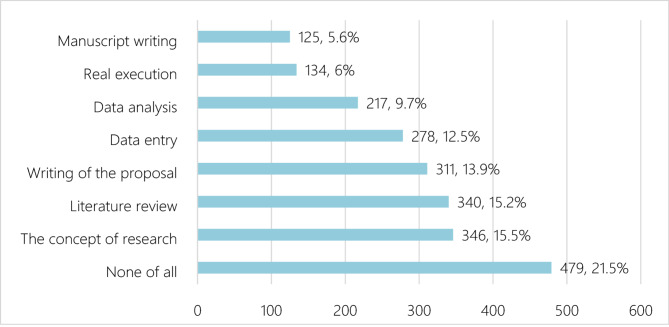
The most common research process that they participated in

Half of the students—50.0%—reported no prior participation in research projects. On average, participants had been involved in approximately 1.22 research projects (SD = 1.96), indicating variability in research engagement. The mean number of publications was 0.79 (SD = 2.03), reflecting a broad range of publication experiences. Additionally, 55.2% of students had not attended any research methodology workshops or training. The average number of poster presentations was 1.03 (SD = 2.16), while the mean number of research-related oral presentations was 1.04 (SD = 2.14). Figures 1 and 2 illustrate the most common type of studies that the students participated in, and the most common research process that they participated in, respectively.

### Perceived barriers toward research

Several key obstacles emerged as prevalent themes. Foremost, an overwhelming 67.6% of students agreed that the lack of timely funding and insufficient financial resources posed a significant hindrance to their research progress. Additionally, 58.1% of students expressed agreement with the barrier of inadequate access to appropriate databases, which can impede the discovery and utilization of relevant information. Another prominent challenge was the lack of access to essential laboratory equipment for conducting research projects, resonating with 64.8% of the students.

Furthermore, 60.4% of students acknowledged the limitation imposed by the lack of access to studies across the country, which restricts the breadth and diversity of research opportunities. Collaboration between research centers was also identified as a significant barrier, with 60.3% of participants agreeing that insufficient cooperation hampers research progress. In addition, 55.9% of respondents highlighted the scarcity of suitable research space as a constraint that affects their ability to conduct research effectively.

### Factors affecting knowledge, attitudes, practices, and burden of barriers toward research

Regarding knowledge score, attending a research methodology course at the university had a significant impact on knowledge, with those who attended having a higher mean score (1.6) compared to those who did not (0.7) (*p* < 0.0011). Anxiety score showed a significant difference based on sex (*p* < 0.0011), with males (mean: 14.7) reporting higher anxiety levels compared to females (mean: 13.7). Attending a research methodology course had a significant impact on positive attitude, with those who attended having a higher mean score (18.1) compared to those who did not (17.3) (*p* < 0.0011). Usefulness for profession score showed a significant difference based on sex (*p* = 0.0031), with females (mean: 24.1) perceiving research as more useful for their profession compared to males (mean: 23.5). University type also had a significant impact, with private university students (mean: 23.7) perceiving research as less useful compared to public university students (mean: 24.1) (*p* = 0.0151) (Table 2).

**Table 2:** Comparisons between knowledge, attitudes, and barriers scores according to sex, university type, and attending research methodology course by Mann–Whitney test:

		Sex			University type			Attending research methodology course at the university		
	Total (*N* = 1123)	Male (*N* = 334)	Female (*N* = 789)	*p*-value	Public (*N* = 643)	Private (*N* = 480)	*p*-value	Yes (*N* = 635)	No (*N* = 488)	*p*-value
**knowledge score**				0.732			0.517			< 0.001
Mean (SD)	1.2 (1.4)	1.3 (1.3)	1.2 (1.4)		1.2 (1.4)	1.3 (1.4)		1.6 (1.5)	0.7 (1.1)	
Range	0.0–7.0	0.0–6.0	0.0–7.0		0.0–7.0	0.0–6.0		0.0–7.0	0.0–5.0	
**Burden of barriers score**				0.877			0.110			0.723
Mean (SD)	78.8 (13.1)	78.9 (12.8)	78.8 (13.2)		79.3 (13.0)	78.1 (13.1)		78.7 (12.7)	78.9 (13.5)	
Range	32.0–96.0	32.0–96.0	32.0–96.0		32.0–96.0	32.0–96.0		32.0–96.0	32.0–96.0	
**difficulty**				0.057			0.799			0.185
Mean (SD)	5.1 (1.6)	5.3 (1.6)	5.1 (1.6)		5.1 (1.7)	5.1 (1.6)		5.1 (1.7)	5.2 (1.6)	
Range	3.0–9.0	3.0–9.0	3.0–9.0		3.0–9.0	3.0–9.0		3.0–9.0	3.0–9.0	
**Relevance to life**				0.324			0.118			0.505
Mean (SD)	8.5 (1.7)	8.4 (1.6)	8.5 (1.7)		8.6 (1.7)	8.4 (1.7)		8.5 (1.8)	8.5 (1.5)	
Range	4.0–12.0	4.0–12.0	4.0–12.0		4.0–12.0	4.0–12.0		4.0–12.0	4.0–12.0	
**Anxiety**				< 0.001			0.833			0.903
Mean (SD)	14.0 (4.2)	14.7 (4.2)	13.7 (4.1)		14.0 (4.2)	14.0 (4.1)		14.0 (4.3)	14.0 (4.0)	
Range	8.0–24.0	8.0–24.0	8.0–24.0		8.0–24.0	8.0–24.0		8.0–24.0	8.0–24.0	
**Positive attitude**				0.373			< 0.001			< 0.001
Mean (SD)	17.6 (3.4)	17.5 (3.5)	17.7 (3.3)		17.9 (3.2)	17.2 (3.6)		17.3 (3.6)	18.1 (3.1)	
Range	7.0–21.0	7.0–21.0	7.0–21.0		7.0–21.0	7.0–21.0		7.0–21.0	7.0–21.0	
**Usefulness for Profession**				0.003			0.015			0.039
Mean (SD)	23.9 (3.3)	23.5 (3.7)	24.1 (3.1)		24.1 (3.2)	23.7 (3.4)		23.8 (3.2)	24.2 (3.4)	
Range	9.0–27.0	9.0–27.0	9.0–27.0		9.0–27.0	11.0–27.0		9.0–27.0	9.0–27.0	

Individuals with previous research project experience and those who have undergone research methodology training or workshops demonstrate significant higher knowledge scores (*p* < 0.001). Moreover, participants who engaged in research projects reported a significantly lower burden of barriers score (*p* < 0.001), indicating fewer obstacles in conducting research. Enrolling in research methodology training or workshops was significantly associated with higher difficulty score (*p* < 0.001). Both previous participation in a research project and research methodology training or workshops were significantly linked to higher “relevance to life” scores (*p* = 0.0031 and *p* < 0.001, respectively) and higher anxiety scores (*p* = 0.0221 and *p* < 0.001, respectively) (Table 3).

**Table 3:** Comparisons between knowledge, attitudes, and barriers scores according to previous participation in a research project and previous enrollment in a research methodology training or workshops by Mann–Whitney test:

		Previous participation in a research project			Previous enrollment in a research methodology training or workshops		
	Total (*N* = 1123)	Yes (*N* = 561)	No (*N* = 562)	*p*-value	Yes (*N* = 503)	No (*N* = 620)	*p*-value
**knowledge score**				< 0.001			< 0.001
Mean (SD)	1.2 (1.4)	1.6 (1.5)	0.9 (1.2)		1.7 (1.6)	0.9 (1.1)	
Range	0.0–7.0	0.0–7.0	0.0–6.0		0.0–7.0	0.0–6.0	
**Burden of barriers score**				< 0.001			0.104
Mean (SD)	78.8 (13.1)	77.5 (13.0)	80.1 (13.0)		78.1 (12.5)	79.4 (13.5)	
Range	32.0–96.0	32.0–96.0	32.0–96.0		32.0–96.0	32.0–96.0	
**difficulty**				0.205			< 0.001
Mean (SD)	5.1 (1.6)	5.2 (1.6)	5.1 (1.6)		5.4 (1.6)	4.9 (1.6)	
Range	3.0–9.0	3.0–9.0	3.0–9.0		3.0–9.0	3.0–9.0	
**Relevance to life**				0.003			< 0.001
Mean (SD)	8.5 (1.7)	8.7 (1.8)	8.4 (1.5)		8.8 (1.8)	8.2 (1.5)	
Range	4.0–12.0	4.0–12.0	4.0–12.0		4.0–12.0	4.0–12.0	
**Anxiety**				0.022			< 0.001
Mean (SD)	14.0 (4.2)	14.3 (4.2)	13.7 (4.1)		14.7 (4.3)	13.4 (4.0)	
Range	8.0–24.0	8.0–24.0	8.0–24.0		8.0–24.0	8.0–24.0	
**Positive attitude**				0.418			0.023
Mean (SD)	17.6 (3.4)	17.6 (3.5)	17.7 (3.3)		17.9 (3.3)	17.4 (3.5)	
Range	7.0–21.0	7.0–21.0	7.0–21.0		7.0–21.0	7.0–21.0	
**Usefulness for profession**				0.062			0.781
Mean (SD)	23.9 (3.3)	23.7 (3.3)	24.1 (3.3)		24.0 (3.2)	23.9 (3.4)	
Range	9.0–27.0	9.0–27.0	9.0–27.0		11.0–27.0	9.0–27.0	

Among the participants, research knowledge scores significantly increased with the academic year (*p* < 0.001). Attitude scores differed across years (*p* = 0.002), peaking in third and fourth years. Research methodology education and project participation rose markedly with academic level (both *p* < 0.001). The number of research projects (*p* = 0.006), poster (*p* = 0.008), and oral presentations (*p* = 0.044) also varied significantly by year, while publication counts showed no significant difference (*p* = 0.558). Workshop attendance increased with seniority (*p* < 0.001) (Table 4).

**Table 4:** Comparison of research knowledge, attitudes, barrier score, and participation across academic years among medical students (*N* = 1123)

	First year (*N* = 102)	Second year (*N* = 234)	Third year (*N* = 233)	Fourth year (*N* = 174)	Fifth year (*N* = 186)	Sixth year (*N* = 194)	Total (*N* = 1123)	*P* value
**Knowledge score (Mean ± SD)**	0.7 (1.3)	0.9 (1.2)	1.1 (1.4)	1.5 (1.5)	1.4 (1.3)	1.8 (1.6)	1.2 (1.4)	< 0.001^1^
**Barrier score (Mean ± SD)**	80.4 (14.3)	78.5 (13.5)	78.8 (12.5)	78.2 (13.5)	77.3 (13.5)	80.3 (11.6)	78.8 (13.1)	0.221^1^
**Attitude score (Mean ± SD)**	69.2 (6.9)	69.2 (7.6)	70.5 (8.3)	70.5 (9.6)	67.6 (9.0)	68.0 (9.3)	69.2 (8.6)	0.002^1^
**Did you study research methodology in your university?**								
Yes	13.0 (12.7%)	64.0 (27.4%)	87.0 (37.3%)	128.0 (73.6%)	162.0 (87.1%)	181.0 (93.3%)	635.0 (56.5%)	< 0.001^2^
No	89.0 (87.3%)	170.0 (72.6%)	146.0 (62.7%)	46.0 (26.4%)	24.0 (12.9%)	13.0 (6.7%)	488.0 (43.5%)	
**Did you participate in a research project before?**								
Yes	28.0 (27.5%)	66.0 (28.2%)	105.0 (45.1%)	95.0 (54.6%)	129.0 (69.4%)	138.0 (71.1%)	561.0 (50.0%)	< 0.001^2^
No	74.0 (72.5%)	168.0 (71.8%)	128.0 (54.9%)	79.0 (45.4%)	57.0 (30.6%)	56.0 (28.9%)	562.0 (50.0%)	
**Number of projects (Mean ± SD)**	1.3 (2.5)	0.8 (1.8)	1.2 (2.0)	1.4 (2.1)	1.3 (1.7)	1.4 (1.7)	1.2 (2.0)	0.006^1^
**Number of publications (Mean ± SD)**	0.9 (2.5)	0.6 (2.0)	0.9 (2.0)	0.9 (2.2)	0.9 (2.0)	0.7 (1.6)	0.8 (2.0)	0.558^1^
**Did you attend a research workshop or training before?**								
Yes	25.0 (24.5%)	66.0 (28.2%)	108.0 (46.4%)	98.0 (56.3%)	106.0 (57.0%)	100.0 (51.5%)	503.0 (44.8%)	< 0.001^2^
No	77.0 (75.5%)	168.0 (71.8%)	125.0 (53.6%)	76.0 (43.7%)	80.0 (43.0%)	94.0 (48.5%)	620.0 (55.2%)	
**Number of poster presentations (Mean ± SD)**	0.9 (2.1)	0.7 (1.7)	1.5 (2.7)	1.0 (1.9)	1.1 (2.3)	0.9 (1.9)	1.0 (2.2)	0.008^1^
**Number of oral presentations (Mean ± SD)**	1.0 (2.4)	0.8 (2.0)	1.4 (2.5)	1.1 (2.0)	1.2 (2.3)	0.8 (1.6)	1.0 (2.1)	0.044^1^

¹Kruskal-Wallis test, ²Pearson Chi-Square Test

The results of the Spearman’s rank correlation analysis revealed a significant positive correlation between age and knowledge score (rho = 0.205, *p* < 0.001), suggesting that older students tend to possess higher levels of knowledge. Additionally, a significant negative correlation between burden of barriers score and Knowledge score (rho = -0.085, *p* = 0.004). Furthermore, higher knowledge score correlate significantly with higher attitude scores (rho = 0.179, *p* < 0.001), suggesting that good knowledge may lead to more positive attitudes (Table 5).

**Table 5:** Spearman’s rank correlation analysis for age, knowledge, attitude, and burden barrier score:

Correlation	Statistics	Interpretation
Age – Total attitude	ρ = -0.030, *p* = 0.319	No significant correlation
Age – Total knowledge	ρ = 0.205, *p* < 0.001	Weak positive correlation, statistically significant
Age – Total barrier	ρ = 0.014, *p* = 0.629	No significant correlation
Total attitude – Total knowledge	ρ = 0.179, *p* < 0.001	Weak positive correlation, statistically significant
Total attitude – Total barrier	ρ = -0.025, *p* = 0.411	No significant correlation
Total knowledge – Total barrier	ρ = -0.085, *p* = 0.004	Weak negative correlation, statistically significant

## Discussion

This cross-sectional study sheds light on the knowledge, attitudes, practices, and perceived barriers to research among undergraduate medical students in Sudan. The results reveal a low level of research knowledge, a generally positive attitude toward research, recognition of its importance, and limited engagement in research activities. Students reported facing major obstacles, including limited funding, restricted access to databases, and a lack of laboratory equipment.

The mean knowledge score observed highlights an alarmingly low level of research knowledge among students, even lower than that reported at Zagreb University, Croatia [15]. The poor knowledge levels may also impact research productivity, potentially contributing to the low number of publications from Sudanese students and the country’s weakened presence in the international academic arena. Moreover, insufficient research knowledge among future medical professionals could undermine their understanding and application of evidence-based practice, with potential consequences for the quality of patient care [16]. Our study found no significant differences in research knowledge between students from public and private universities. This suggests that individual factors—such as personal motivation, self-initiative, and engagement—may play a more critical role in shaping students’ research competencies than the type of institution alone. Notably, our findings indicate that students with previous research exposure—such as attending a research methodology course or participating in a research project—achieved significantly higher knowledge scores. This underscores the important role of practical research experience in strengthening students’ understanding. Similar findings were reported in a study from Turkey [17], which also demonstrated improved research knowledge among students with prior involvement in research activities.

Furthermore, in our study, research knowledge scores increased significantly with both advancing academic year and age, consistent with findings from Jordan and Pakistan [18, 19]. This trend may reflect greater cumulative exposure to research opportunities, enhanced access to faculty mentorship, and institutional requirements in many medical schools for senior students to complete and submit a research thesis as part of their graduation criteria [20]. Together, these findings highlight the importance of early and sustained integration of research training within medical education.

Despite the low knowledge scores, students expressed a positive attitude toward research and a strong recognition of its relevance to their future careers. This aligns with studies conducted in Egypt, Saudi Arabia, and Cape Town, where the majority of students acknowledged the significance of research participation for their professional development [21–24]. This favorable attitude provides a solid foundation for cultivating a stronger research culture within the medical community. Interestingly, our study also found that higher knowledge scores were significantly correlated with more positive attitudes toward research, suggesting that improved knowledge may foster more favorable perceptions—similar to findings from a previous Egyptian study [9]. Attitude scores varied across academic years, with the highest levels observed among third- and fourth-year students. Several previous studies have shown that students’ attitudes toward research tend to improve as they progress through medical school [23, 25].

The study presented mixed findings regarding research practices among students. Although the majority had not participated in research projects, an average participation rate of 1.22 projects indicates that a portion of students is engaging to some extent. However, the considerable variability in research experience, reflected by an average of 0.79 publications, suggests that many students lack the necessary support and experience to convert participation into concrete research outputs. It is concerning that half of the students had never attended a research methodology workshop, a finding consistent with observations from other developing countries like Nepal, where fewer than one-third of students reported attending such workshops [26]. Without sufficient training, students are unlikely to make meaningful research contributions, reinforcing the urgent need for comprehensive programs to equip them with essential research skills. The observed disparity in poster and oral presentation experiences, with averages of 1.03 and 1.04 respectively, may indicate gaps in the curriculum, compelling students to rely largely on self-directed learning to acquire research competencies. Research methodology education and participation in research projects increased markedly with advancing academic level. This finding is consistent with a study from Saudi Arabia, which reported a direct relationship between students’ engagement in medical research and their academic year [23]. In Sudanese medical schools, completing a thesis is a mandatory requirement for graduation [20], and most students undertake it during their final or pre-final year. This likely encourages greater attendance at research methodology workshops and increased involvement in research activities during the later stages of medical education.

Several barriers were identified as limiting students’ participation in research, with lack of timely funding emerging as the most commonly reported obstacle. The intensive demands of the medical curriculum, coupled with frequent clinical examinations, often compel students to prioritize other academic commitments over research activities. Similar challenges were noted in studies from Saudi Arabia, Pakistan, and Mumbai [4, 19, 27, 28]. Two-thirds of the students cited limited funding as a major barrier, a finding that mirrors those reported in other studies [13, 28]. Financial limitations not only hinder access to essential resources but also restrict the breadth and quality of research projects. Another significant barrier was limited access to comprehensive research databases, consistent with findings from Iran and Egypt [29–31]. This underscores the need to improve students’ access to research literature and tools. Furthermore, nearly two-thirds of the students reported inadequate laboratory equipment as a barrier, a challenge also highlighted in previous research [30, 32]. Addressing these infrastructural deficiencies is vital to enable students to engage in and produce high-quality research. Participants who engaged in research projects reported significantly lower barrier scores, suggesting they faced fewer obstacles in conducting research. Conversely, enrollment in research methodology training or workshops was associated with higher perceived difficulty. This may indicate that gaining theoretical knowledge through workshops increases awareness of the challenges involved, creating a perception that research is more difficult. However, actual participation in a research project may help demystify the process, making it feel more manageable in practice.

A key strength of this study is its large and diverse sample size, which included medical students from both public (57.3%) and private (42.7%) universities across Sudan. Given that the actual number of public and private medical schools is approximately equal [33], this reflects near-equal institutional representation. The gender distribution (70.3% female, 29.7% male) also closely aligns with national estimates [34], indicating strong gender representativeness. These factors enhance the generalizability of the findings. Additionally, the study used a validated and reliable questionnaire adapted from previous research, ensuring the credibility of the measured variables. Conducting the survey online allowed wide accessibility during the ongoing conflict, overcoming logistical challenges and reaching students across different regions. Furthermore, the comprehensive assessment of knowledge, attitudes, practices, and barriers provides a holistic understanding of the research landscape among Sudanese medical students.

The main limitations of this study include potential sampling bias due to convenience sampling and the cross-sectional design, which restricts causal inference and tracking changes over time. The use of self-reported data may also introduce recall and social desirability biases. Future research should adopt more representative sampling strategies, longitudinal designs, and qualitative methods to gain deeper insights into students’ research experiences.

## Conclusion

This study highlights both the potential and challenges facing undergraduate medical students in Sudan regarding research engagement. While students demonstrate a positive attitude toward research and recognize its importance for their future careers, their knowledge and practical involvement remain limited. Several barriers to research participation were identified, including a lack of time, funding, access to databases, and laboratory equipment. These findings mirror those reported in other developing countries, indicating broader systemic gaps in research education and infrastructure.

To strengthen research engagement among medical students, it is important to incorporate research training into the undergraduate curriculum and promote mentorship programs that guide students through research activities. Expanding opportunities for active research participation, organizing workshops to build basic research skills, and improving access to research resources such as databases and laboratory facilities are also needed. Furthermore, increasing institutional support and funding for student research can help create an environment that nurtures research culture and encourages students to contribute meaningfully to scientific advancement.

## Data Availability

“The datasets used and/or analysed during the current study are available from the corresponding author on reasonable request.”

## Abbreviations

SPSS: Statistical Package for the Social Sciences
STROBE: Strengthening the Reporting of Observational Studies in Epidemiology
